# Robot-assisted deep brain stimulation with intraoperative CT imaging and frameless registration module: a new gold-standard?

**DOI:** 10.1007/s00701-025-06581-w

**Published:** 2025-06-13

**Authors:** Gonzague Defrance, Philippe Domenech, Johan Pallud, Marc Zanello

**Affiliations:** 1 Department of Neurosurgery, GHU , Hôpital Sainte-Anne, Paris, France; 2 Université de Paris Cité, Paris, France; 3 UMR 1266, IMA-BRAIN, Institute of Psychiatryand , INSERM, Neurosciences of Paris, Paris, France; 4 Department of Psychiatry, Service Hospitalo-Universitaire, Groupe Hospitalier Universitaire (GHU) Paris Psychiatrie Et Neurosciences, Site Sainte-Anne, Paris, France; 5 Commissariat À L’énergie Atomique Et Aux Énergies Alternatives (INSERM-CEA), Cognitive Neuroimaging Unit, NeuroSpin Institut National de La Santé Et de La Recherche Médicale, Gif-Sur-Yvette, France; 6 Institut de Neuromodulation, Groupe Hospitalier Universitaire (GHU) ParisCentre Hospitalier Sainte-AnneUniversité Paris Cité, Psychiatrie Et NeurosciencesPôle Hospitalo-Universitaire 15, Paris, France

**Keywords:** Robotic surgical procedures, Monitoring, Intraoperative, Diagnostic techniques, Neurological, Image processing, Computer-assisted, Psychosurgery, Parkinson disease, Essential tremor

## Abstract

**Background:**

Although stereotactic frame-based techniques remain widely used for deep brain stimulation (DBS) implantation, robot-assisted procedures have demonstrated improved accuracy, enhanced precision, and reduced operative time. Our team has over two decades of experience in robot-assisted surgery and intraoperative imaging.

**Method:**

We detail our current surgical workflow for DBS implantation, combining the Neuromate robot (Renishaw), the NeuroLocate frameless registration module, and intraoperative cone-beam CT imaging using the O-Arm system (Medtronic).

**Conclusion:**

This approach provides a safe, efficient, and reproducible alternative to traditional methods, supporting its broader adoption in modern functional neurosurgery.

**Supplementary Information:**

The online version contains supplementary material available at 10.1007/s00701-025-06581-w.

## Relevant surgical anatomy

The described surgical technique –i.e. the combined use of a surgical robot and intraoperative mobile cone-beam CT– is applicable regardless of the indication and anatomical target selected for Deep Brain Stimulation (DBS).

## Description of the technique

**Fig. 1:**
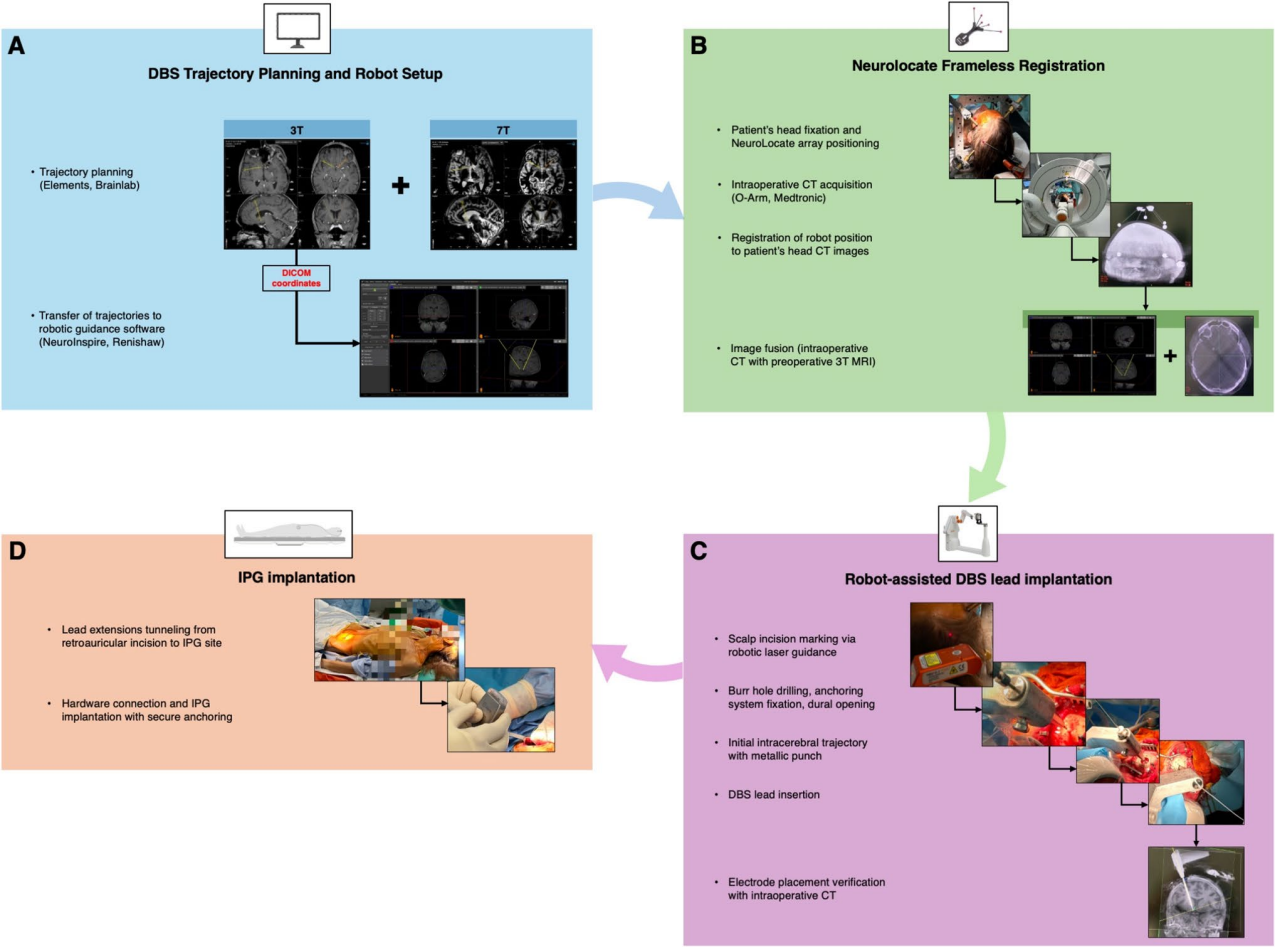
Workflow of robot-assisted DBS implantation using frameless registration and intraoperative imaging. **A** DBS trajectory planning is performed using an ad hoc software: Elements (Brainlab, Munich, Germany). Dedicated 3T and 7T MRI sequences are used to define optimal target and trajectories. The trajectories are then exported – based on DICOM coordinates in a referential 3T anatomical image – to NeuroInspire software (Renishaw, New Mills, UK), used for robotic guidance. **B** Frameless registration is performed with the NeuroLocate module (Renishaw). The patient’s head is secured in a stereotactic frame (Leksell G frame or Talairach frame), and the NeuroLocate array is positioned close to the skull. An intraoperative cone-beam CT scan is acquired using the O-Arm system (Medtronic, Minneapolis, MN). The robot is registered to the patient’s position based on automatic recognition of the array spheres. This intraoperative CT is then fused with the preoperative MRI data, allowing the robot to navigate within the anatomical space defined by the MRI with real-time correspondence to the patient’s position in the operating room. **C** Robot-assisted DBS lead implantation includes successively scalp incision marking via laser guidance, scalp incision, robot-guided pre-drilling and subsequent burr hole realization, thinning of the outer cranial vault and screwing of the anchoring device, dural opening, realization of an initial intracerebral trajectory using a metallic punch, lead implantation, confirmation of electrode positioning with an intraoperative CT before securing the lead with the anchoring device and funneling of the distal end toward the retroauricular region. **D** IPG implantation involves subcutaneous tunneling of extensions from the retroauricular region to the IPG site (subclavicular or abdominal), followed by connection and secure anchoring of the IPG

Figure 1 and Video describe the surgical procedure.

Robot-assisted DBS implantation is performed under general anesthesia without intraoperative microelectrode recordings (MER). Even though MER can be used to refine electrode placement, they do not preclude from trajectory errors and significantly prolong surgical duration [1].

### Preoperative imaging and planning

Preoperative imaging includes:

1. 3T MRI, under general anesthesia for patients with movement disorders, including sequences such as 3D T2-FLAIR, susceptibility-weighted imaging (SWAN), MPRAGE, and white matter-nulled acquisition (F-GATIR). Diffusion tensor imaging (DTI) with at least 50 diffusion-encoding directions enables deterministic tractography reconstruction. The MRI protocol depends on the desired target.
2. High-resolution 7T MRI including MP2RAGE, MPRAGE, SWI, and T2-FLAIR sequences, tailored for enhanced visualization of DBS targets.

**Fig. 2:**
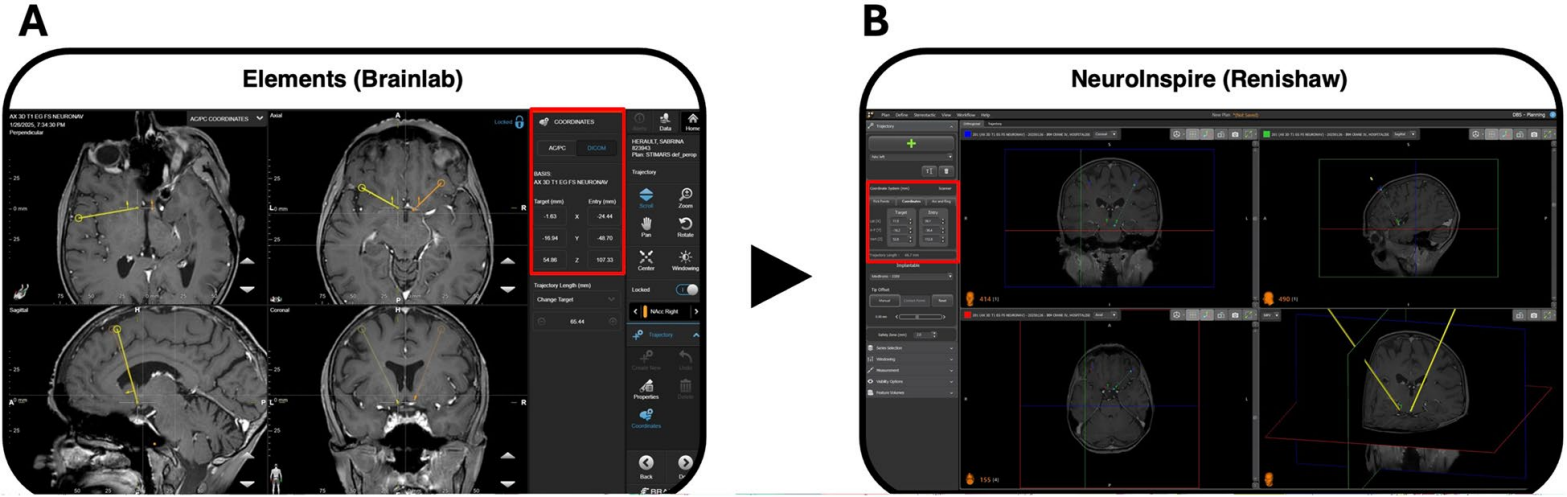
Preoperative stereotactic planning of DBS trajectories for robot-assisted procedure. **A** Trajectory planning is performed using Elements software (version 1.6.2, Brainlab AG, Munich, Germany). A referential T1 isovoxel image, acquired on the eve of surgery with a 3T MRI scan, serves as the anatomical space for coordinate definition. Trajectories are defined, depending on the target, using a combination of indirect targeting, direct visualization from coregistered 3T and 7T sequences, and tractography-derived anatomical landmarks (not shown). In this case, bilateral trajectories targeting the nucleus accumbens were planned for a patient with severe, pharmacoresistant anorexia nervosa. Trajectories DICOM coordinates (highlighted in the red box) are given in the referential T1 space. **B** These coordinates are then imported into NeuroInspire software (Renishaw, New Mills, UK), which is used for robotic guidance. The same 3T T1 referential image is also imported and later fused with the intraoperative 3D cone-beam CT (O-Arm, Medtronic, Minneapolis, MN). This fusion allows the robot to navigate the preoperatively defined trajectories within the patient’s actual intraoperative anatomical space with millimetric accuracy

MRI acquisitions are coregistered, and DBS trajectories are planned using Elements software (version 1.6.2, Brainlab, Munich, Germany). Trajectories must position at least two electrode contacts within the target, avoiding sulci and intracranial vessels. DICOM coordinates in the patient’s preoperative T1 anatomical space are transferred to NeuroInspire software (Renishaw, UK) for robotic guidance (Fig. 2).

### Patient positioning and frameless registration

**Fig. 3:**
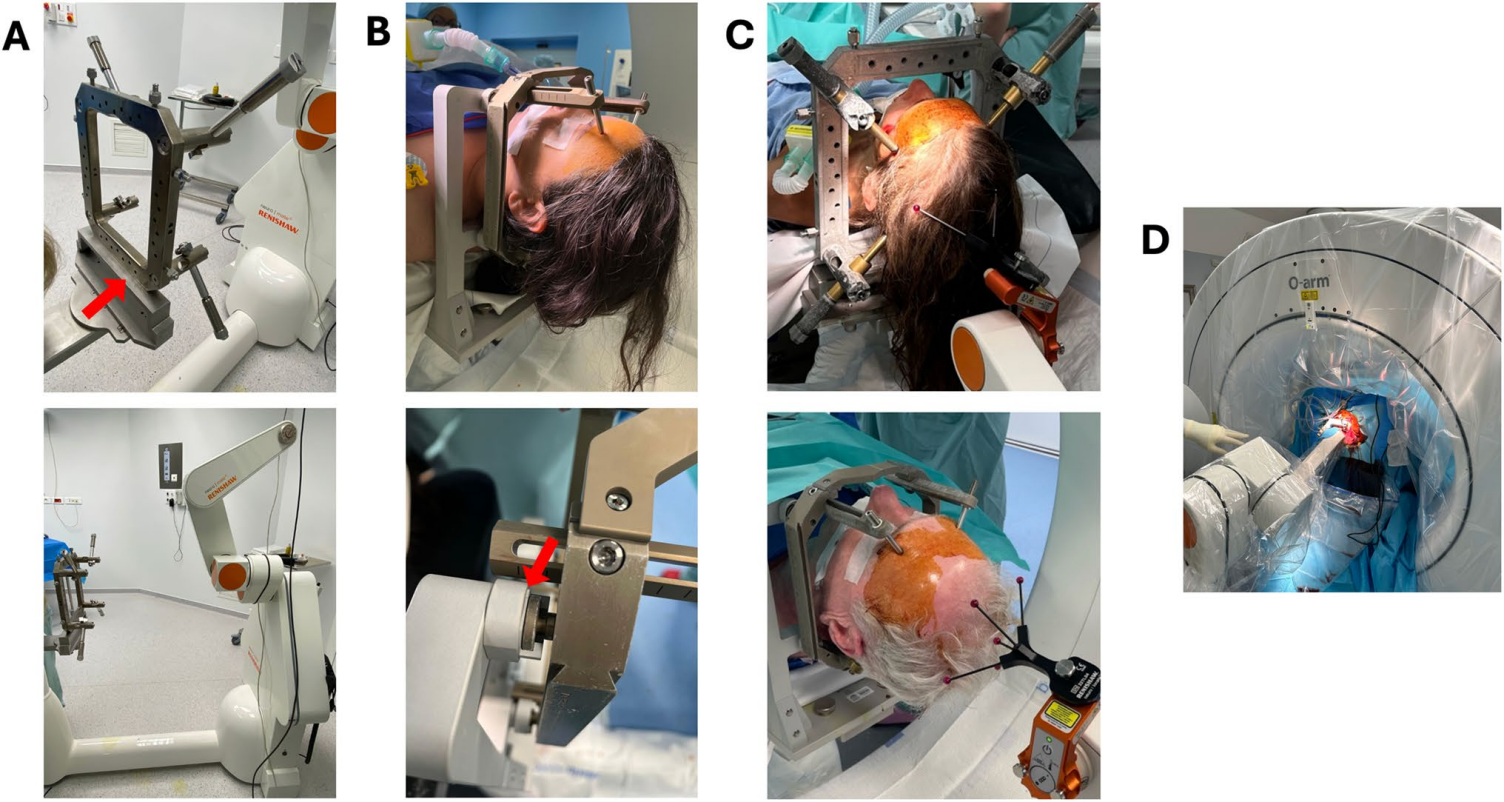
Patient positioning and surgical setup for robot-assisted DBS implantation. **A–C** Head fixation systems used in our robot-assisted procedure. The surgical technique is compatible with both the Talairach frame (Dixi, Besançon, France) – historically used at Sainte Anne Hospital –, and the Leksell G frame (Elekta, Stockholm, Sweden), each secured to the Neuromate robotic arm (Renishaw, New Mills, UK). **A** The Talairach frame is fixed to the robotic arm using a dedicated attachment system (upper), which locks onto the base screws of the frame (red arrow). The lower panel provides a wider view of the Talairach frame secured to the Neuromate. **B** The Leksell G frame is secured using a dedicated attachment system that consist of three anchoring points (upper). The lower panel shows a top-view close-up of one of the anchoring points (red arrow). **C** Final intraoperative positioning with either frame in place—Talairach (upper) and Leksell G (lower)—and the NeuroLocate array mounted on the robotic arm and placed near the patient’s head for accurate frameless registration. **D** Intraoperative surgical setup illustrating robot positioning and intraoperative O-Arm cone-beam CT imaging system (Medtronic, Minneapolis, MN) draped and positioned around the patient’s head, allowing real-time volumetric imaging for trajectory verification and electrode placement control

The patient’s head is secured in either a Talairach frame base (Dixi, Besançon, France) (Fig. 3A) or a Leksell G frame base (Elekta, Stockholm, Sweden) (Fig. 3B), both compatible with the 5-degree-of-freedom Neuromate robot (Renishaw, New Mills, UK) (Fig. 3C). Frameless registration is performed intraoperatively using the NeuroLocate array (Renishaw), comprising five precisely spaced ruby spheres attached to the robotic arm near the patient’s head.

Intraoperative 3D imaging with an O-Arm cone-beam CT scanner (Medtronic, Minneapolis, MN) allows real-time volumetric images (Fig. 3D). NeuroInspire software uses initial intraoperative CT to recognize the NeuroLocate spheres and uses their known spatial configuration to define the patient’s head position relative to the robotic coordinate system. A safety step is performed by checking the agreement of the robot’s arm positioning with a test trajectory before proceeding. Fusion of preoperative MRI data and intraoperative CT enables robotic navigation within the anatomical space defined by the MRI with real-time correspondence to the patient’s position in the operating room. This workflow provides millimetric accuracy without requiring skin fiducials or a stereotactic frame [2].

### Surgical procedure and electrode placement

The robotic system is aligned with the planned trajectories, using a laser pointer to mark entry points onto the scalp. After minimal hair shaving, antisepsis, and sterile tool holder placement, a Kirschner pin inserted along the first trajectory verifies accurate alignment through a second intraoperative CT scan. Once this verification completed, the operative field is fully draped. A first 2,5-mm pilot hole is drilled along the trajectory axis under robotic guidance. This first hole guides the drilling of a classical 14 mm burr-hole. The outer cranial table is thinned to minimize skin prominence of the anchoring device that is then secured to the skull with self-drilling screws and checked for correct closure.

A limited dural incision and gentle cortical coagulation are performed and the robot is advanced as close as possible to the burr hole, ensuring it does not interfere with anchoring device closure. An initial intracerebral trajectory up to the target is realized using a 1.5- or 1.8-mm metallic punch, followed by DBS lead insertion through a dedicated 1.4-mm barrel. Accuracy of electrode placement relative to preoperative planning is confirmed by another intraoperative CT. The lead anchoring device is secured, and the procedure is repeated contralaterally. Distal ends of the leads are then secured with protective caps, funneled subcutaneously toward the retroauricular area, and cranial incisions are closed.

### Internal Pulse Generator (IPG) Implantation

The O-Arm and the robot are removed from the surgical field. The patient’s head is released from the fixation frame. Distal ends of the leads can be gently advanced subcutaneously toward the retroauricular region by scalp massage. A second sterile preparation exposes the retroauricular area and planned IPG site (subclavicular or abdominal). A subcutaneous pocket is created for the IPG, with extensions tunneled from the retroauricular region to the IPG site, passing by the midline. Retromuscular placement of the IPG is considered in thin patients to optimize device concealment and comfort. Extensions are connected respectively with DBS lead and IPG. Impedance checks are performed and the IPG is securely anchored to prevent device displacement (Twiddler’s syndrome). Incisions are closed meticulously.

The day following surgery, a postoperative CT scan confirms final electrode placement and the absence of any radiological complication.

## Indications

The present surgical technique—combining stereotactic planning with robotic guidance based on frameless registration and intraoperative 3D imaging—is applicable to a wide range of stereotactic neurosurgical procedures, such as stereoelectroencephalography (SEEG) [8], third ventriculostomy and stereotactic biopsies [3, 6, 10]. Its precision make it particularly well-suited for DBS interventions requiring millimetric accuracy [7]. DBS is classically performed for movements disorders, such as Parkinson Disease, Essential Tremor, Dystonia. Nevertheless, indications arise such as DBS for drug-resistant epilepsy, drug-resistant pain or psychiatric disorders, highlighting the relevance of robot-assisted surgical approach that ensures precision, reproducibility, and procedural efficiency.

## Limitations

Equipment availability, costs, and the need for a trained surgical team constitute primary limitations. However, the technology’s multi-procedural versatility across neuroendoscopy [4], SEEG [8], and biopsies [3, 6, 10] can justify broader institutional adoption. Our workflow relies on general anesthesia without MER, underscoring the importance of high-quality imaging and accurate coregistration. Nonetheless, awake clinical testing and MER remain feasible with this workflow.

## How to avoid complications

Complication avoidance requires rigorous adherence to each procedural step. Preoperative trajectory planning is best performed using dedicated stereotactic software (e.g. Brainlab Elements) that allows precise visualization of cortical and subcortical anatomy as well as vascular structures. Trajectories reviewed independently by two neurosurgeons enhance planning robustness. We recommend using high-resolution imaging —3T and 7T MRI— with sequences acquired under general anesthesia in patients with movement disorders, to reduce motion artifacts.

Special caution is required during image coregistration, as 7T MRI is prone to geometric distortions, particularly at air–tissue interfaces and outer edges of the head. These distortions can lead to misalignment of 7T images with 3T and intraoperative CT images [9]. To mitigate this risk, we recommend ROI–based coregistration centered on deep brain structures which are less affected by distortion, and careful manual verification of all image fusions. Frameless registration and robotic guidance should rely on anatomical 3T MRI as reference space. 7T MRI should be used only as secondary images fused onto this reference space to guide trajectory planning.

Beyond these considerations, adherence to key principles of stereotactic surgery is essential. The patient’s head should be maintained in a neutral position, partially flexed and dural opening should remain limited to reduce cerebrospinal fluid leakage and the risk of brain shift. The tunneling of extension cables should be performed in a single step, passing through the midline to IPG site. The IPG must be securely anchored to prevent displacement and ensure proper charging in the case of rechargeable systems. It should be positioned within 2 cm of the skin surface, with a retromuscular placement considered in the case of very thin patients – for instance patients suffering for anorexia nervosa—for aesthetic outcome and to preserve skin. The retroauricular incision must be placed away from areas where eyeglass arms may rest.

## Specific information for the patient

Patients undergoing robot-assisted DBS under general anesthesia should be informed that the procedure does not involve intraoperative stimulation testing stimulation. Clinical outcomes are similar to those achieved with awake, frame-based procedures, and implantation precision is equal or superior [5, 7].

Optimal symptom control may take weeks to months to be achieved, especially in psychiatric indications, which might require up to 18 months to reach full efficacy. Patients must be aware of multiple small incisions (cranial entry, retroauricular, IPG site) and potential hardware-related risks, which are minimized by secure anchoring techniques. Clear communication regarding MRI compatibility, programming adjustments, and future IPG replacements is essential.

## 10 key points summary

1. Robot-assisted DBS with frameless registration and intraoperative 3D cone-beam CT present advantages over frame-based procedures, including high mechanical accuracy, reproducibility, and procedural efficiency.
2. Trajectory planning requires optimal preoperative imaging. Combining 3T MRI—performed under general anesthesia for patients with movement disorders—and complementary 7T MRI ensures optimal anatomical targeting.
3. Planning is best performed using a dedicated software and should be independently reviewed by two experienced neurosurgeons.
4. Frameless registration via the NeuroLocate array and intraoperative cone-beam CT imaging allows the robotic arm to precisely navigate within the anatomical space defined by preoperative 3T MRI data, providing real-time positional accuracy.
5. 7T MRI imaging, despite high anatomical resolution, should serve as complementary data due to potential geometric distortions and should not be used for primary robotic registration or navigation.
6. Creating a pilot hole under robotic guidance facilitates precise burr hole placement. Thinning the surrounding outer cranial table minimizes prominence through the skin and visibility of the anchoring device.
7. An initial trajectory using a rigid needle (diameter similar to the DBS lead) guides subsequent lead implantation. The robotic arm should be positioned as close as possible to the cortical surface to minimize mechanical deviation without obstructing anchoring device closure.
8. The implantation site of the IPG should be decided in agreement with the patient. Abdominal placement is typically better tolerated in younger or thin patients
9. The IPG should be securely anchored subcutaneously, or retromuscularly in thin patients, to minimize displacement risks (e.g., Twiddler’s syndrome), and to enhance patient comfort and cosmesis.
10. Patients undergoing robot-assisted DBS under general anesthesia without intraoperative stimulation testing should be informed that clinical outcomes are comparable to those achieved with “awake” frame-based procedures, with equal or superior implantation precision. Additionally, clear communication regarding postoperative programming adjustments, MRI compatibility considerations, and long-term device management remains essential for achieving optimal clinical outcomes.

## Supplementary Information

Below is the link to the electronic supplementary material.

ESM 1 (MP4 313 MB)

## Data Availability

No datasets were generated or analysed during the current study.
